# Maternal separation produces alterations of forebrain brain-derived neurotrophic factor expression in differently aged rats

**DOI:** 10.3389/fnmol.2015.00049

**Published:** 2015-09-01

**Authors:** Qiong Wang, Feng Shao, Weiwen Wang

**Affiliations:** ^1^Department of Psychology, Peking UniversityBeijing, China; ^2^Key Laboratory of Mental Health, Institute of Psychology, Chinese Academy of SciencesBeijing, China

**Keywords:** maternal separation, brain-derived neurotrophic factor (BDNF), medial prefrontal cortex (mPFC), hippocampus

## Abstract

Early life adversity, such as postnatal maternal separation (MS), play a central role in the development of psychopathologies during individual ontogeny. In this study, we investigated the effects of repeated MS (4 h per day from postnatal day (PND) 1–21) on the brain-derived neurotrophic factor (BDNF) expression in the medial prefrontal cortex (mPFC), the nucleus accumbens (NAc) and the hippocampus of male and female juvenile (PND 21), adolescent (PND 35) and young adult (PND 56) Wistar rats. The results indicated that MS increased BDNF in the CA1 and the dentate gyrus (DG) of adolescent rats as well as in the DG of young adult rats. However, the expression of BDNF in the mPFC in the young adult rats was decreased by MS. Additionally, in the hippocampus, there was decreased BDNF expression with age in both the MS and non separated rats. However, in the mPFC, the BDNF expression was increased with age in the non separated rats; nevertheless, the BDNF expression was significantly decreased in the MS young adult rats. In the NAc, the BDNF expression was increased with age in the male non-maternal separation (NMS) rats, and the young adult female MS rats had less BDNF expression than the adolescent female MS rats. The present study shows unique age-differently changes on a molecular level induced by MS and advances the use of MS as a valid animal model to detect the underlying neurobiological mechanisms of mental disorders.

## Introduction

Adverse early life events are considered to be risk factors for the development of psychiatric diseases (Walker and Diforio, 1997; Ellenbroek and Cools, 1998; Marais et al., 2008; Réus et al., 2011). In rats, maternal separation (MS), which deprives pups of their mothers, has often been used as a model for early life adversity (Hall, 1998; Marco et al., 2009). MS has been demonstrated to induce behavioral and cognitive abnormalities, such as increased depressive and anxiety-like behaviors (Marais et al., 2008; Jia et al., 2009; Rentesi et al., 2010) and prepulse inhibition (PPI) deficits (Ellenbroek and Cools, 1998, 2002). MS has also been shown to decrease new born cells in the hippocampus and the granular cell number in the dentate gyrus (DG) of juvenile and adult rats (Mirescu et al., 2004; Oreland et al., 2010; Hulshof et al., 2011); these findings suggest that MS can affect the neuroplasticity of rats.

Brain-derived neurotrophic factor (BDNF) is a member of the neurotrophin family (Hyman et al., 1991) and exerts a wide range of functions, such as maintaining neuronal survival, structure, growth, and differentiation and promoting synaptic plasticity of learning and memory (Fumagalli et al., 2007; Pillai and Mahadik, 2008). BDNF has also been implicated in the neurobiological mechanisms of psychiatric diseases (Weickert et al., 2003; Schneider et al., 2011; Favalli et al., 2012). BDNF expression in multiple brain regions is sensitive to adverse life experiences. For example, our lab has reported that adolescent social isolation affects BDNF levels in the medial prefrontal cortex (mPFC), Nucleus accumbens (NAc) and hippocampus of adult rats (Han et al., 2011; Meng et al., 2011). Several studies have reported the effects of MS on BDNF levels in different brain areas; however, the results have been inconsistent. For example, MS increased the BDNF level in the hippocampus of adult rats (Greisen et al., 2005; Faure et al., 2007), reduced the BDNF levels in the PFC, hippocampus and striatum of mice (Ognibene et al., 2008) or had no change with respect to the BDNF levels in the PFC and hippocampus (Réus et al., 2011). These discrepancies may be resulted from the different experimental procedures, species and strains adopted in these studies.

Furthermore, other studies have indicated that the developmental period of animals may be another important factor for MS effects. For example, Roceri et al. (2004) reported that MS produced a short-term-up-regulation of the BDNF level in the hippocampus and PFC on postnatal day (PND) 17 and a reduction of BDNF expression in the PFC in adulthood. Kuma et al. (2004) conformed that MS decreased the BDNF mRNA expression on PND 16 and increased the BDNF mRNA expression on PND 30 and 60 in the hippocampus of rats, and there was no significant difference between MS and non-maternal separation (NMS) rats on PND 20. Although these studies mentioned above suggested the developmental factors of MS effects, to date, there is not any studies that investigated the effects of MS on forebrain BDNF expression in juvenile, adolescent and young adult rats systematically.

In the present study, we aimed to investigate the effects of repeated MS (4 h/day from PND 1–21) on the BDNF expression levels in the mPFC, NAc and hippocampus in juvenile (PND 21), adolescent (PND 35) and young adult (PND 56) rats. The three brain regions chosen in this study were based on the close functional relationships with BDNF activity of them.

## Material and Methods

### Animals

Male and female Wistar rats were obtained from the Academy of Chinese Military Medical Science. All of the animals were housed on a 12 h light/12 h dark cycle (lights on at 7:00 a.m.) and with free access to food and water. The environmental conditions was kept constant (ambient temperature 22°C). All experimental procedures were performed in strict accordance with the guidelines of the National Institutes of Health Guide for the Care and Use of Laboratory Animals (NIH Publications No. 80–23) and approved by the Institutional Animal Care and Use Committee (IACUC) of Peking University.

### Maternal Separation

The protocol of MS were adapted from previous studies (Li et al., 2013; Wang et al., 2015). The male and female rats were mated to produce litters that consisted of 8–12 pups. After birth, the pups were randomly divided into two groups: the MS (MS group, 48 pups) and the NMS (NMS group, 48 pups). MS was performed on MS group while the NMS group was undisturbed from PND 1–21. Each group had 24 male and 24 female pups. During the separation, the pups in MS group were separated from their mothers for 4 h (10:00–14:00) per day from PND 1–21 and maintained on heated sawdust (29 ± 1°) separately from their littermates. The dams of the pups in MS group were left in the home cage during the separation. The pups in NMS group remained in their home cage with their mothers and littermates during the 4 h separation. After weaning at PND 21, 16 MS (8 males and 8 females) and 16 NMS (8 males and 8 females) animals were sacrificed for the BDNF measurement by immunohistochemistry (IHC). The rest of rats were reallocated to different cages (4 rats per cage). Then, at PND 35, another 16 MS (8 males and 8 females) and 16 NMS (8 males and 8 females) animals were sacrificed for the BDNF measurement by IHC. At PND 56, the last 16 MS (8 males and 8 females) and 16 NMS (8 males and 8 females) animals were sacrificed for the BDNF measurement by IHC.

### Immunohistochemistry

This procedure IHC has been described in previous studies (D’andrea et al., 2001; Xavier et al., 2005; Han et al., 2011; Meng et al., 2011). Briefly, the rats were anesthetized of intraperitoneal (ip) administration with chloral hydrate (400 mg/kg) and perfused with phosphate buffered saline (PBS, 0.01 M) followed by 4% paraformaldehyde dissolved in PBS. The brain regions of interest (mPFC: 5.70–2.70 mm from bregma; NAc: 2.70–0.70 mm from bregma; hippocampus: −1.30 to −5.30 mm from bregma; Paxinos and Watson, 2006) were dissected on ice using a rat brain mold and post-fixed by 4% paraformaldehyde for 6 h. Then the brain samples were dehydrated (3 × 30 min 70%, 90%, 96%, 100% ethanol and Roti-Histol) and embedded in paraffin. Next, the paraffin which containing the brain samples were cut into sections (4 μm) using a microtome (Leica 235), then the sections were pasted onto slides and dried (30 min, 58°C) on a heating plate. After washing in 0.05 M PBS (3 × 2 min), the sections were put into citrate buffer solution and heated in microwave oven. Afterwards, the slides were blocked (10% goat serum and 1% BSA dissolved in 0.01 M PBS, 20 min at room temperature, RT) and incubated in a first rabbit anti-BDNF polyclonal IgG (1:200, Santa Cruz Biotechnology, overnight at 4°C). After washing in 0.05 M PBS (3 × 5 min, RT), the sections were incubated with a secondary goat anti-rabbit IgG (1:1000, Santa Cruz Biotechnology, 1 h at RT), and incubated in an avidin–biotin–peroxidase complex (1 h). Finally, the sections were dehydrated by serial alcohol rinsing, dewaxed in dimethyl benzene, and cover-slipped.

### Quantification and Statistical Analyses

The slides were viewed and photographed using a light microscope (Olympus BX-51), and the images were analyzed using a software (Image-pro plus 6.0). The BDNF levels were estimated by counting all of the BDNF-positive cells present in two serial sections interspaced by 4 μm in the middle of the mPFC, NAc and hippocampus. The areas of the mPFC, NAc and the CA1, CA2/3 and DG of the hippocampus were measured, and the number of units per 1 mm^2^ was calculated bilaterally per rat. For the analysis, the cell counts were averaged into a single score for each rat.

All of the data are shown as the mean ± standard error of the mean (SEM). The analyses were performed using the SPSS 16 software. The IHC results were analyzed using a multivariate analysis of variance (MANOVA). The comparisons with two and three groups were analyzed using Student’s *t*-test and a one-way ANOVA followed by least significant difference (LSD) *post hoc* tests, respectively. The significance level was defined as *p* < 0.05.

## Results

### Effects of MS on the BDNF Expression in the Hippocampus

**Figure 1:**
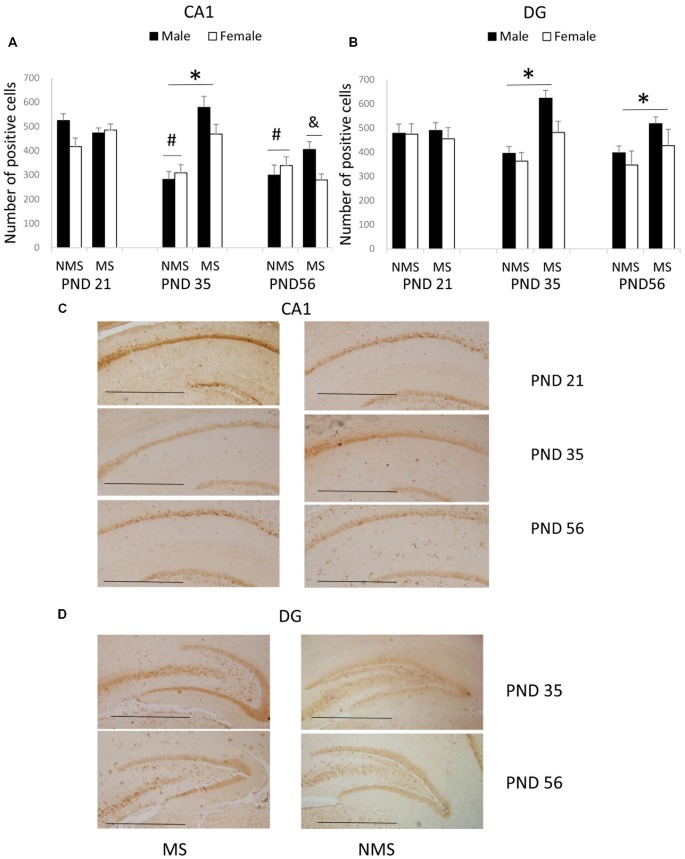
**Effects of Maternal separation (MS) on the brain-derived neurotrophic factor (BDNF) expression in the hippocampus, including the effects of MS on the BDNF expression at different ages in CA1 (A) and dentate gyrus (DG) (B) and representative immunohistochemistry (IHC) figures of CA1 (C) and DG (D).** The results are expressed as the mean ± S.E.M. (*compared with PND 35 NMS rats, *p* < 0.05; ^#^compared with PND 21 NMS rats, *p* < 0.05; &compared with PND 21 and PND 35 MS rats, *p* < 0.05). Scale bar = 250 μm.

The results of the BDNF expression in the CA1 were summarized in Figure 1. The results showed that there were significant main effects of MS (*F*_(1,84)_ = 7.987, *p* = 0.006) and age (*F*_(2,84)_ = 7.421, *p* = 0.001), but not gender. The interaction between MS and age was significant (*F*_(2,84)_ = 5.385, *p* = 0.006), whereas the other interactions were not significant. Further analysis (*t*-test) indicated that MS increased the BDNF expression in the CA1 in the PND 35 rats (*t*_(30)_ = 4.035, *p* < 0.001; Figure 1A). A one-way ANOVA revealed that there was a significant difference among the three ages in both the NMS and MS groups (NMS: *F*_(2,45)_ = 8.211, *p* = 0.001; MS: *F*_(2,45)_ = 5.099, *p* = 0.010). The *post hoc* (LSD) comparisons revealed that in the NMS group, the PND 35 and PND 56 rats had significantly less expression compared with the PND 21 rats; in the MS group, the PND 56 rats had significantly less expression compared with the PND 21 and PND 35 rats (Figure 1A).

In the CA2/3, the overall effects of MS, age and gender were not significant; all of the interactions were also not significant.

The BDNF expression in the DG was summarized in Figure 1B. MS resulted in an overall increased BDNF protein expression in the DG (*F*_(1,84)_ = 7.741, *p* = 0.007). The overall effects of age and gender were not significant, and all of the interactions were also not significant. Further analysis (*t*-test) showed that MS significantly increased the BDNF expression in the DG in the PND 35 and PND 56 rats (PND 35: *t*_30_ = 2.350, *p* = 0.026; PND 56: *t*_30_ = 2.169, *p* = 0.038).

### Effects of MS on the BDNF Expression in the mPFC

**Figure 2:**
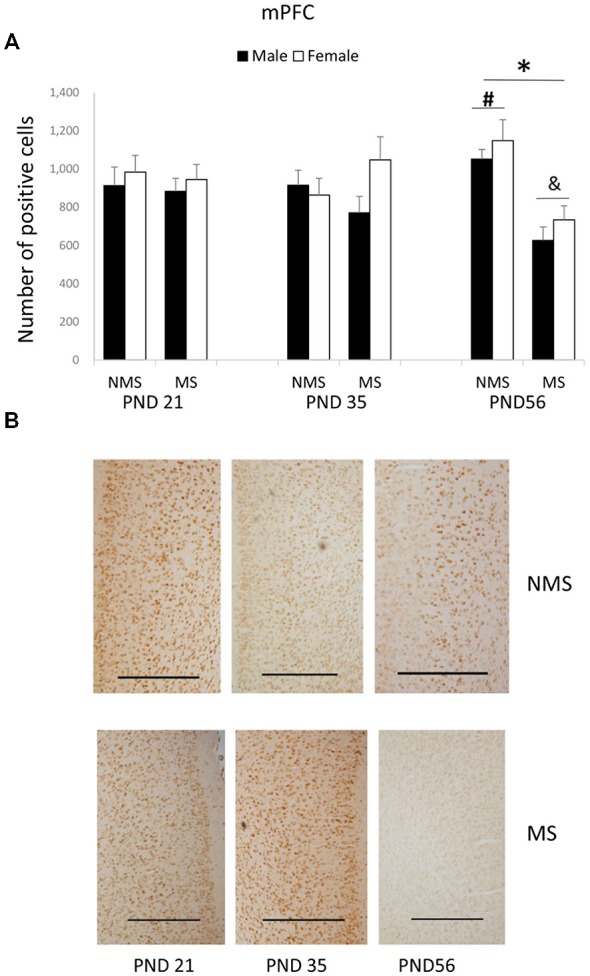
**Effects of MS on the BDNF expression in the mPFC, including the effects of MS on the BDNF expression at different ages (A) and representative IHC figures (B).** The results are expressed as the mean ± S.E.M. (*compared with PND 56 NMS rats, *p* < 0.05; ^#^compared with PND 35 NMS rats, *p* < 0.05; &compared with PND 21 and PND 35 MS rats, *p* < 0.05). Scale bar = 500 μm.

The BDNF expression in the mPFC for each group was summarized in Figure 2. MS significantly decreased the BDNF expression in the mPFC (*F*_(1,84)_ = 4.006, *p* = 0.005). The influence of age and gender on the expression of BDNF was not significant. The interaction between MS and age was significant (*F*_(2,84)_ = 7.749, *p* = 0.001), whereas the other interactions were not significant. Further analysis (*t*-test) indicated that MS reduced the BDNF expression in the mPFC in the PND 56 rats (*t*_(30)_ = 5.350, *p* < 0.001; Figure 2A). A one-way ANOVA revealed that there was a significant difference among the three ages in both the NMS and MS groups (NMS: *F*_(2,45)_ = 3.238, *p* = 0.049; MS: *F*_(2,45)_ = 4.607, *p* = 0.015). The *post hoc* (LSD) comparisons revealed that in the NMS group the PND 56 rats had significantly increased expression compared with the PND 35 rats; in the MS group the PND 56 rats had significantly less expression compared with the PND 21 and PND 35 rats (Figure 2A).

### Effect of MS on the BDNF Expression in the NAc

**Figure 3:**
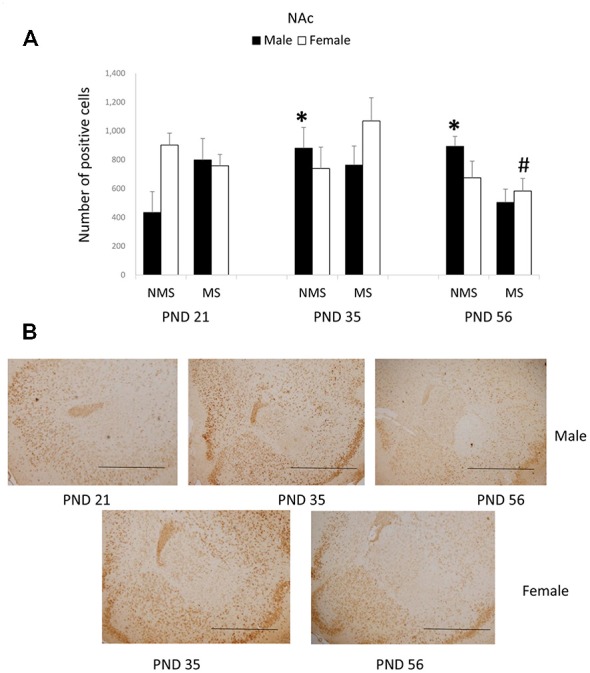
**Effects of MS on the BDNF expression in the Nucleus accumbens (NAc), including the effects of age on the BDNF expression in different gender rats (A) and the representative IHC figures (B).** The results are expressed as the mean ± S.E.M. (*compared with PND 21 male NMS rats, *p* < 0.05; ^#^compared with PND 35 female MS rats, *p* < 0.05). Scale bar = 500 μm.

In the NAc, the overall effects of MS, age and gender were not significant; however, there was a significant interaction among MS, age and gender (*F*_(2,84)_ = 4.475, *p* = 0.014). The other interactions were not significant. Further analysis (one-way ANOVA) revealed that for the male rats there was a significant difference among the three ages in the NMS, but not in the MS groups (NMS: *F*_(1,22)_ = 4.378, *p* = 0.026; MS: *F*_(1,22)_ = 1.613, *p* = 0.223). However, for the female rats there was a significant difference among the three ages in the MS, although not in the NMS groups (NMS: *F*_(1,22)_ = 0.970, *p* = 0.395; MS: *F*_(1,22)_ = 4.564, *p* = 0.023). The *post hoc* (LSD) comparisons revealed that in the male NMS group the PND 21 rats had significantly less expression compared with the PND 35 and PND 56 rats (Figure 3A); in the female MS group the PND 56 rats had significantly decreased BDNF expression compared with the PND 35 rats (Figure 3B).

## Discussion

The current findings showed that repeated MS has fundamental effects on BDNF protein expression in the forebrain of juvenile, adolescent and young adult male and female rats. MS increased BDNF expression in hippocampus of rats but decreased it in the mPFC. BDNF expression in the CA1 was decreased with age. However, in the mPFC, the increased expression of BDNF with age in non-separated rats was reversed in MS rats. The effect of gender on BDNF expression was only found in the NAc.

Firstly, our results suggested that there were different effects of MS on BDNF expression in the mPFC, hippocampus and NAc. MS increased in the hippocampus of adolescent and young adult rats and decreased in the mPFC of young adult rats. Similarly, one previous study reported that MS produced a short-term-up-regulation of BDNF expression in hippocampus and PFC, which was measured on PND 17, and a reduction of BDNF expression in the PFC in adulthood (Roceri et al., 2004). Our results indicated that MS did not affect the expression of BDNF in NAc. The alike results were also found in our previous study in adult rats (Xue et al., 2013). These findings reminded us that MS may affect BDNF expression differently in the mPFC, hippocampus and NAc.

Since BDNF was closely related to both neural plasticity and cytoarchitecture, are the influences of MS has on neural plasticity and cytoarchitecture in mPFC and hippocampus similar? Previous study reported that MS (3 h per day from PND 2–14) increased the hippocampal neurogenesis which was assessed by using BrdU and DCX, meanwhile, the histone methylation at the *BDNF* IV promoter and the expression of BDNF were also increased in hippocampus (Suri et al., 2013). MS also increased CREB and BDNF levels and hippocampus progenitor proliferation in hippocampus (Nair et al., 2007). Another study demonstrated that MS (2 h per day from PND 1–12) decreased the dendritic length and dendritic spine density of the neuron in PFC (Monroy et al., 2010). MS (24 h on PND 9) also induced the reduction in the thickness of PFC and of the density of NeuN-immunolabeled neurons in PFC of rats (Aksić et al., 2013). These studies on cytoarchitecture and neural plasticity supported our findings about changes on BDNF levels in PFC and hippocampus induced by MS.

In addition, existing studies have shown that the inconsistency of MS effects was also reflected in cognitive function, such as the spatial learning and reversal learning in Morris water maze (MWM). Our recent study reported that repeated MS (4 h per day from PND 1–21) increased swim distance in spatial learning and decreased escape latency in reversal learning of MWM in adolescent and early adult rats (Wang et al., 2015). MS could induce the impairment of spatial learning of MWM in adolescent (Frisone et al., 2002) and adult rats (Garner et al., 2007), and also could enhance the performance in reversal learning of MWM in adult rats (Lehmann et al., 1999). Hippocampus, which was a key brain region of memory, played an important role in cognitive function in MWM. Specifically deleted BDNF in hippocampus impaired the spatial learning in MWM (Heldt et al., 2007). PFC was also closely associated with spatial working memory in MWM (Xing et al., 2012). These behavioral and BDNF results suggested that differences among cognitive abnormalities of MS animals may be related to the diverse changes in hippocampus and mPFC, but more researches are still needed.

Secondly, as we have noted above, the developmental period of animals may be another important factor for the MS effects. The present study investigated the effects of MS on BDNF expression in the hippocampus and mPFC of juvenile, adolescent and young adult rats. Our results found that MS did not change the BDNF expression in the CA1 and DG of PND 21 rats, but increased the BDNF expression in the CA1 and DG of PND 35 rats, as well as in the DG of PND 56 rats. Similarly, another previous study indicated that MS (3 h per day from PND 10–15) decreased the expression of BDNF mRNA in the hippocampus of PND 16 rats and increased the expression of BDNF mRNA in the hippocampus of PND 30 and 60 rats. What’s more, it was reported that there was no significant difference between MS and mother-reared control rats on PND 20 (Kuma et al., 2004). These findings were consistent with the results in the present study.

Thirdly, regarding the effects of MS on male and female rats, the present study found that the expression of BDNF in the mPFC and hippocampus was not significantly different between the male and female rats. These results reminded us that the expression of BDNF, which was affected by MS, was not different between the male and female rats. Marco et al. (2013) also found that MS decreased the expression of BDNF in the PFC and hippocampus both in male and female adolescent rats, but MS increased the expression of glial fibrillary acidic protein (GFAP) in male adolescent rats, not in female rats. However, our results showed that between the MS and NMS rats of different ages the effect of gender on BDNF expression in the NAc was different. In the male NMS rats BDNF expression was increased with age. A recent study reported that male juvenile rats (PND 26–28) exhibited significantly elevated basal BDNF expression in the NAc compared with their male adult (4 months) counterparts (Perreault et al., 2013). These results were different with our findings and this discrepancy may be because we did not observe data at 4 months after birth. In the female MS group the BDNF expression of the young rats was decreased compared with the adolescent rats. No comparable result was reported in the previous study.

Fourthly, the present study also found that the expression of BDNF in the CA1 was decreased with age in the NMS rats, and MS did not affect a change with age. A previous study reported that the expression of BDNF in the hippocampus changed with age: the expression of BDNF increased after birth and it reached the highest level in the second week after birth. After that, the expression of BDNF gradually declined with age (Silhol et al., 2005). These findings, consistent with our results, revealed the effect of age on the process of BDNF neural-development in the brain.

More importantly, the present study found that compared with juvenile and adolescent rats, the BDNF expression in the mPFC of young adult rats was significantly affected by repeated MS. The highest BDNF expression in the NMS young adult rats was reversed to the lowest expression of the MS rats. These results suggested that the increase of BDNF expression in the mPFC for young adult rats may be stopped and reversed by MS, and no comparable result was reported in previous studies. However, our lab had reported that repeated MS (4 h per day from PND 1–21) improved reversal learning of the MWM in young adult rats. For NMS rats, compared with juvenile or adolescent rats, young adult rats had significantly decreased cognitive flexibility; for MS rats, MS significantly improved reversal learning of young adult rats (Wang et al., 2015). Most previous studies had reported a positive relationship between BDNF expression in the PFC and cognitive function (Bredy et al., 2007; Sakata et al., 2013). However, a recent report found that stress facilitated reversal learning of mouse, and ventromedial prefrontal cortex (vmPFC) lesions mimicked this effect of stress, but this enhanced reversal learning of mouse induced by stress was prevented by BDNF infusion into the vmPFC (Graybeal et al., 2011), which in part supported our findings. Altogether, the relationship between cognitive function and BDNF expression in the mPFC requires further investigation.

Epigenetics research also confirmed the influence of early life adversity on BDNF expression in brain. Roth et al. (2009) demonstrated that early maltreatment increased the methylation of *BDNF* DNA and it reduced the BDNF expression in PFC of adult rats. Furthermore, many studies have confirmed that MS led to hyperactivity of hypothalamic-pituitary-adrenal (HPA) axis (Plotsky and Meaney, 1993; Lehmann et al., 2002; Lippmann et al., 2007). MS reduced the level of glucocorticoid type-2 and corticotropin-releasing hormone type-1 receptor (CRH1) mRNA in hippocampus. MS also impaired the memory function and decreased the expression of glucocorticoid in hippocampus of rats (Llorente et al., 2011). These biochemical changes may contribute to the neurobiological foundation of behavioral and cognitive alterations induced by MS, which should be further detected in our future research.

## Conclusion

These present findings suggest that repeated MS induced different types of forebrain neurobiological changes in juvenile, adolescent, and young adult rats, revealing the varying patterns of BDNF expression along with age in different brain regions and indicating that the influence of gender was only embodied in NAc not mPFC or hippocampus. The present study provided new evidence for the study of behavioral and neuro-biochemical alterations induced by adverse early life event. Considering the close relationship between human BDNF and early life adversity, these findings have potential clinical implication for treating mental disorders.

## Author Contributions

FS designed the research; QW performed the research and acquired the data; QW, FS and WW interpreted and analyzed the data; and QW, FS and WW drafted, revised and wrote the paper.

## Conflict of Interest Statement

The authors declare that the research was conducted in the absence of any commercial or financial relationships that could be construed as a potential conflict of interest.
